# Prenatal Development and Function of Human Mononuclear Phagocytes

**DOI:** 10.3389/fcell.2021.649937

**Published:** 2021-04-08

**Authors:** Mohi Miah, Issac Goh, Muzlifah Haniffa

**Affiliations:** ^1^Biosciences Institute, Newcastle University, Newcastle upon Tyne, United Kingdom; ^2^Department of Dermatology and NIHR Newcastle Biomedical Research Centre, Newcastle Hospitals NHS Foundation Trust, Newcastle upon Tyne, United Kingdom; ^3^Wellcome Sanger Institute, Hinxton, United Kingdom

**Keywords:** human mononuclear phagocytes, developmental immunology, monopoiesis, prenatal human mononuclear phagocytes, organoids, single cell transcriptomics, immunobiology, sc-RNA seq

## Abstract

The human mononuclear phagocyte (MP) system, which includes dendritic cells, monocytes, and macrophages, is a critical regulator of innate and adaptive immune responses. During embryonic development, MPs derive sequentially in yolk sac progenitors, fetal liver, and bone marrow haematopoietic stem cells. MPs maintain tissue homeostasis and confer protective immunity in post-natal life. Recent evidence – primarily in animal models – highlight their critical role in coordinating the remodeling, maturation, and repair of target organs during embryonic and fetal development. However, the molecular regulation governing chemotaxis, homeostasis, and functional diversification of resident MP cells in their respective organ systems during development remains elusive. In this review, we summarize the current understanding of the development and functional contribution of tissue MPs during human organ development and morphogenesis and its relevance to regenerative medicine. We outline how single-cell multi-omic approaches and next-generation *ex-vivo* organ-on-chip models provide new experimental platforms to study the role of human MPs during development and disease.

## Introduction

The mononuclear phagocyte (MP) system includes macrophages, monocytes, and their precursors, classified based on their morphology, function, and origin, with macrophages initially assumed to be differentiated monocytes (van Furth and Cohn, 1968). Dendritic cells (DCs) were discovered later. Defined by their probing morphology and ability to activate naïve T-cells, they were incorporated into the MP system (Steinman and Cohn, 1973; Collin and Bigley, 2018).

In 1882, Elie Metchnikoff suggested that macrophages participate in the maintenance of tissue integrity and homeostasis. This required macrophages to be able to discriminate between the self and non-self, recognize tissue damage, and sense invading pathogens (Tauber, 2003). Since then, multitudes of studies have continuously refined and redefined our understanding of MP function. The roles MPs play in post-natal life have been studied in depth; however, the functional heterogeneity of the MP system during human gestation is still poorly understood (Ginhoux and Jung, 2014; Hoeffel and Ginhoux, 2015; Hoeffel et al., 2015). Recent studies have shown MPs are present from 6 post-conception weeks (PCW) in a human pathogen-free *in utero* environment (Popescu et al., 2019; Park et al., 2020), thus suggesting that MPs may play a non-canonical role – a role unrelated to protective immunity – in organogenesis and tissue morphogenesis during development.

In this review, we summarize the consensus view on human MP development, outline the diverse functions of MPs in prenatal life and compare them to their roles in post-natal life. We also explore the use of organoids and organ-on-chip (OoC) models to interrogate MP function *ex vivo*. We further provide a web portal of manually curated MP markers and associated protein interaction networks stratified by species, organ, and developmental time^1^.

## Development of the Human MP System

Human embryonic haematopoiesis occurs in several transient waves. Generation and differentiation of haematopoietic progenitors begin in the human yolk sac (YS), giving rise to the first myeloid cells appearing in the human YS at 2–3 PCW and to macrophage populations (Ginhoux and Jung, 2014; Hoeffel and Ginhoux, 2015; Bian et al., 2020).

**FIGURE 1:**
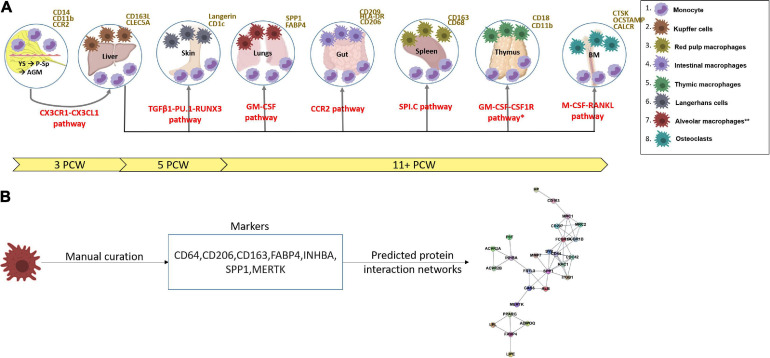
**(A)** A scheme showing the functional timeline and earliest characterization of mononuclear phagocytes and the pathways involved (red) from the yolk sac into the fetal liver (Migliaccio et al., 1986; Ginhoux and Jung, 2014; Hoeffel and Ginhoux, 2015; Bian et al., 2020), thymus, spleen, gut, skin, lungs (alveolar macrophages denoted with ** as they arise post-natally) detected from 11 post-conception weeks (Audiger et al., 2017), and bone marrow. Arrows show the journey of circulating monocytes before tissue infiltration. Surface markers of monocytes and tissue residing mononuclear phagocytes are in yellow. *Data from mouse studies – may not have been verified in human studies. YS, yolk sac; P-Sp, para-aortic-splanchnopleura; AGM, aorta-gonad-mesonephros; BM, bone marrow; CS, Carnegie stage; PCW, post-conception weeks. Figures were produced with assistance from Biorender.com. **(B)** A schematic showing how markers for cell states were manually curated and used to compute predicted protein interaction networks. All markers and predicted networks curated are available on an interactive online portal: (https://developmentcellatlas.ncl.ac.uk/MPS_development_review).

Definitive haematopoiesis then follows in the human aorta-gonad-mesonephros (AGM) from 3 to 4 PCW (CS12), characterized by haematopoietic stem cells (HSCs) formed from the haemogenic endothelium (Migliaccio et al., 1986; Ginhoux and Jung, 2014; Hoeffel and Ginhoux, 2015). These HSCs rapidly enter the circulation and seed the fetal liver (FL), which produces the first population of granulocyte-monocyte progenitors (GMPs) and blood monocytes at 4–5 PCW/CS15 (Hoeffel and Ginhoux, 2015; Hoeffel et al., 2015; Figure 1A). In humans, MPs arise from YS progenitors and the AGM-derived HSCs that seed the haematopoietic organs, including the FL; the FL then serves as the main haematopoietic organ during embryonic and early fetal development (<20 PCW) (Ginhoux and Jung, 2014; Haniffa et al., 2015; Hume et al., 2019; Popescu et al., 2019). After birth, adult bone marrow (BM)-derived monocytes can also give rise to macrophages (Hume et al., 1985).

### Monocytes

Monocytes comprise a remarkably plastic population circulating through the blood to surrounding tissues where they differentiate into macrophages or monocyte-derived dendritic cells (mo-DCs).

Three types of monocytes have been observed in adult humans: CD14^++^CD16^–^ classical monocytes, CD14^+^CD16^++^ non-classical monocytes, and CD14^+^CD16^+^ human intermediate monocytes (Ziegler-Heitbrock et al., 2010). The closest equivalent of human CD14^++^CD16^–^ and CD14^+^CD16^++^ monocytes in mice are Ly6C^+^ classical monocytes and Ly6C^–^ non-classical monocytes, respectively. Murine Ly6C^–^ non-classical monocytes have been shown to differentiate from circulating Ly6C^+^ monocytes and patrol the vascular system (Ginhoux and Jung, 2014).

Comparison of human fetal and post-natal monocytes has shown that both fetal and adult populations show a high expression of myeloid and monocyte surface markers CD11b, CD11c, CCR2, and CX3CR1 (Krow-Lucal et al., 2014). Upon interferon γ (IFN- γ) stimulation, post-natal monocytes upregulate antigen presentation genes. Conversely, fetal monocytes upregulate genes involved in innate antimicrobial responses to evade activating adaptive immunity, which could cause anti-self/anti-maternal rejection (Krow-Lucal et al., 2014).

### Macrophages

Macrophages have been observed both morphologically and transcriptionally from the earliest wave of mouse YS haematopoiesis and as early as 6 PCW/CS19 in human YS and decidua (Banaei-Bouchareb et al., 2006; Menassa and Gomez-Nicola, 2018; Vento-Tormo et al., 2018; Popescu et al., 2019). Hofbauer cells, observed from 2.5 PCW, are used to describe any fetal-derived placental macrophage that resides within the placental villous core, amnion, and chorionic larvae (Vento-Tormo et al., 2018; Thomas et al., 2020). Hofbauer cells isolated from the human placenta express CD14, CD163, and secrete anti-inflammatory TGFβ and IL-10, which is suggestive of immune-suppressive and pro-vasculo/angiogenic functions (Johnson and Chakraborty, 2012).

Tissue-resident macrophages (TRM) are perfectly adapted to their resident tissues and have been named based on anatomical location, protein, and transcriptional signatures (Guilliams et al., 2014). The surface markers of macrophages include those shared with monocytes (CD14, CD16, CD68, and CCR5), general macrophage program markers (C1QC and VEGF), and tissue-specific markers, such as VCAM1 for fetal Kupffer cells and PPARγ for alveolar macrophages (Asada et al., 2004; Ginhoux and Jung, 2014; Spivia et al., 2014; Hoeffel and Ginhoux, 2015). TRMs can be long-lived and self-renewing following prenatal seeding but can also be replaced by circulating monocytes (McGovern et al., 2014; Scott et al., 2016; Bajpai et al., 2018; MacParland et al., 2018).

### DCs

Major human dendritic cell (DC) subsets include plasmacytoid DCs (pDC), conventional DC1 (cDC1) and DC2 (cDC2), monocyte-derived DCs (moDC), and Langerhans cells (LCs) (Dzionek et al., 2000; Collin and Bigley, 2018). Although human DC origin has been attributed to BM-derived HSCs, fetal DCs have been observed as early as 6 PCW, suggesting that FL HSCs may also generate DCs (Popescu et al., 2019). Compared to adult DCs, fetal DCs possess an immature phenotype but can induce allogeneic T-cell proliferation upon culture (McGovern et al., 2015). Their presence during early development is attributed to ensuring tolerogenic responses to self and maternal antigens (McGovern et al., 2015).

Post-natally, DCs function to sense pathogens and activate the adaptive immune system (Collin and Bigley, 2018). DCs can arise from both myeloid and lymphoid progenitors (Notta et al., 2016), with studies showing monocyte participation in the DC pool following inflammation (Segura et al., 2013; Tamoutounour et al., 2013).

## Functional Diversification of MPs in Prenatal Life

The distribution of MPs in prenatal organs is carefully coordinated with key timelines of specific organ development, allowing for the continuous survival and development of the fetus (Krow-Lucal et al., 2014; McGrath et al., 2015). Discoveries, including MP origin from progenitors to their post-natal immunological and repair functions, have overshadowed the important roles they play during development and in organogenesis (Hoeffel and Ginhoux, 2015; Hoeffel et al., 2015).

The functional absence of the MP system is embryonically lethal, which shows the importance of MPs for survival during development (De Groote et al., 2014; Rojo et al., 2019). We and others have shown that macrophages, monocytes, and DCs are present from 6 PCW of human life in a relatively pathogen-free *in utero* environment (Popescu et al., 2019; Park et al., 2020). This raises the hypothesis that MPs may play an important role in tissue modeling and homeostasis in addition to immunity during early prenatal life. Below, we synthesize the literature on MP function in the BM, lymphoid, and non-lymphoid barrier organs during human development.

All MP markers and predicted protein interaction are available via an interactive web portal: (see text footnote 1). Markers were stratified by species, organ, cell type, and developmental time. Each set of markers per cell type were submitted as a string protein query using the RCY3 (v3.12) and the STRINGDB module in cytoscape (v3.8). The top 10 interactions per marker with a Stringdb confidence score of >0.9 were embedded in a network plot with nodes representing proteins and edges representing interactions (Figure 1B).

### Bone Marrow

Osteoclasts, the TRMs of the BM, are identified by protein-based surface markers: CTSK, CALCR, SIGLEC15, ACP5, DCSTAMP, OCSTAMP, and TNFRSF11A (Weivoda et al., 2020). Osteoclasts differentiate from common myeloid progenitors via cytokine-dependent signaling involving M-CSF (macrophage-CSF/CSF-1) and receptor-activated NF-κB ligand (RANKL) (Atkins et al., 2003; Tokunaga et al., 2020). They function to assist with the clearance/resorption of bone tissue and are critical in the maintenance, repair, and remodeling of the skeleton (Park et al., 2017).

Osteopetrotic (CSF-1 deficient) mice have excessive bone deposition, deformed bone marrow cavities, and massively altered haematopoiesis. This is due to impaired cell fusion in the absence of CSF-1 required to form osteoclasts (Rojo et al., 2019). *Tnfrsf11a^*cre*^;Csf1r^*fl/fl*^* mice are phenotypically similar to CSF1R-deficient mice, presenting with decreased TRMs, impaired tooth eruption, misshapen skulls, and shorter long bones. However, in contrast to CSF1R-deficient mice, HSCs colonize the bone marrow post-natally in *Tnfrsf11a^*cre*^;Csf1r^*fl/fl*^* mice, leading to the development of osteoclasts, but the prenatally determined deformities persist throughout life (Jacome-Galarza et al., 2019). This suggests that prenatal precursors provide functions required for tooth eruption, skull shape, and long bone development prenatally, whilst post-natal HSC-derived osteoclasts function to maintain bone mass.

Human osteoclasts promote the formation of HSC niches whilst BM macrophages determine haematopoietic egress through the phagocytosis of cells not expressing the “don’t eat me” signaling CD47 ligand (Sacchetti et al., 2007; Witt et al., 2018). Erythroblastic island macrophages in the human BM are in contact with BM erythroblasts to support erythropoiesis. They contribute to heme synthesis and iron recycling (Leimberg et al., 2008), erythropoietin (EPO) feedback sensing (Sawada et al., 1989; Lifshitz et al., 2010), and expression of cytokines, including insulin-like growth factor (IGF) and bone morphogenetic protein (Sawada et al., 1989; Liu et al., 2015), which promote erythropoiesis. Decreased MPs in the BM affects the HSC niche and induces HSC mobilization into the blood due to niche collapse, resulting in a decline in erythro-/haematopoiesis due to bone endosteal niche disruptions (Kaur et al., 2017).

A study on adult human BM DCs showed reduced activity of canonical DC-functionalities when compared to matched DCs in the peripheral blood (van Leeuwen-Kerkhoff et al., 2018). For example, BM-derived cDC2s were less able to upregulate T-cell stimulatory molecules like CD80 upon TLR-triggering when compared to peripheral blood cDC2s (van Leeuwen-Kerkhoff et al., 2018). The BM niche was concluded to be primarily a DC developmental location. Murine BM studies have shown the contribution of DCs in the regulation of haematopoiesis: ablation of murine BM cDCs resulted in HSC mobilization into peripheral blood to transiently lodge into other haematopoietic organs such as the spleen. BM cDC ablation in the mice also led to a loss of BM macrophages, increased BM vascular permeability, and the expansion of BM endothelial cells, which are required for haematopoietic regulation (Zhang et al., 2019). These studies demonstrate the need for MPs in the BM niches during development and how they coincide with post-natal functions to maintain skeletal and BM haematopoietic niches.

### Lymphoid Organs

#### Liver

Kupffer cells, identified by CD163, VCAM1, and CLEC5A in humans (Gonzalez-Dominguez et al., 2015), are the TRMs of the liver, lining the sinusoids. Kupffer cells assist in the proliferation and enucleation of erythroblasts as well as in iron recycling to facilitate erythropoiesis in the FL (Pourcher et al., 2011; Palis, 2016). Definitive erythropoiesis occurs in the human FL and requires HIF2A and EPO expression for progenitor survival. This process is MYB dependent and relies on transcriptional regulators, such as Sox6 and Bcl11A, that down-regulate embryonic (Gower 1 – ζ_2_ε_2_, Gower 2 – α_2_ε_2_) and fetal globin expression (α_2_γ_2_) (Cantu et al., 2011; Bjurström et al., 2016). At the stage of active FL haematopoiesis, macrophages can migrate from the sinusoids to the parenchyma to form erythroblastic islands consisting of a central macrophages surrounded by erythroblasts (Li et al., 2019). The central macrophages express VCAM1, CD163, and EPOR to mediate interactions with early erythroid cells and EPO, stimulating erythroblast enucleation, proliferation, and differentiation (Li et al., 2019; Popescu et al., 2019).

A non-canonical function of human Kupffer cells is to prevent the pathogenic accumulation of lipids in the liver. Peroxisome proliferator-activated receptor gamma (PPARγ) was identified as an important regulator of macrophage activator programs linked to the fatty acid oxidation function of Kupffer cells (Scott et al., 2016; Luo et al., 2017). PPARγ also regulates pro-proliferative interleukin (IL)-4 driven programs (basophil recruitment) during damage to assist during liver generation/regeneration (Daniel et al., 2018). These studies demonstrate a common non-canonical function between human fetal and post-natal Kupffer cells in coordinating erythropoiesis and restoring damaged tissue during fetal development and post-natal liver regeneration.

Like adult DCs, human FL DCs can migrate to lymph nodes and initiate T-cell proliferation in response to toll-like receptor (TLR) ligation. Additionally, it was observed that after TLR ligation, FL cDC2s show markedly reduced TNFα cytokine production when compared to adult DCs to mediate immunosuppressive responses during gestation (McGovern et al., 2017).

#### Thymus

During early human gestation, CD45^+^ early lymphoid progenitors (ELP) have been reported to colonize the fetal thymus from the FL and BM and give rise to plasmacytoid and conventional DC subsets and T-cells (Patel et al., 2009; Park et al., 2020). T-cells undergo positive and negative selection during development. Double positive CD4^+^/CD8^+^ cells that do not recognize MHCs on thymic stromal cells and single positive cells that respond to self-antigens are eliminated by apoptosis. Human DCs have been reported to mediate recognition and clearance of negatively selected cells, whilst thymic epithelial cells (TECs) have been shown to play an essential role in positive selection (Hu et al., 2015). Deactivated DCs characterized in the fetal thymus revealed gene expression programs adapted for this role, with AIRE supporting the negative selection of T-cells, and chemokines (CCL17, CCL19, and CCL22), enabling recruitment of CD4 T-cells and Tregs to the thymic medulla (Park et al., 2020). Thus, DC depletion in the thymus could lead to an increased propensity for autoimmunity reactions due to a lack of negative selection (Audiger et al., 2017; Park et al., 2020).

The early thymic MP system is composed of Mac1^+^ (CD11b/CD18) thymic macrophages (TMs) observable from 8 PCW (Park et al., 2020), but little is known of their prenatal function. During murine development, TMs phagocytose apoptotic thymocytes, assisting with the clearance of negatively selected cells, carrying out DNA fragmentation via DNase-II-dependent degradation in lysosomes (Kawane et al., 2003). DNase-II knockout mice with impaired macrophage function during development displayed reduced brain, kidney, and thymic size due to the accumulation of undigested apoptotic cell debris within phagocytes (Kawane et al., 2003). A study on E14.5 mice thymi demonstrated CD4^+^/CD11b^+^ macrophages exhibiting phagocytosis of apoptotic thymocytes (Esashi et al., 2003). RUNX1 knockout mice have impaired macrophage development and display impaired thymic development, including the accumulation of double-negative thymocytes (Putz et al., 2006).

#### Spleen

Splenic TRMs originate from the FL and fetal BM. The spleen functions to clear blood-borne pathogens and acts as an early haematopoietic organ during development, bridging erythro-/haematopoiesis between the FL and BM. It is divided into red pulp and white pulp fractions separated by the marginal zone. The macrophages in each zone have specific functions and interactions (Hoeffel and Ginhoux, 2015).

Human red pulp macrophages (RPM) form a vast network required for the uptake of senescent red blood cells and iron homeostasis. They express CD163 and CD68 and are SPI-C dependent. RPMs selectively upregulate SPI-C expression, driving HMOX1 expression, which encodes the essential heme recycling enzyme, Heme Oxygenase 1 (HO-1) (Haldar et al., 2014; Nagelkerke et al., 2018). Splenic monocytes may also express SPI-C when induced by free heme from red-blood-cell degradation, generating new RPMs (Haldar et al., 2014). Prenatally, RPMs localize in splenic cords and assess the condition of erythrocytes. CD47 expression on erythrocytes inhibits phagocytosis via interaction with the signal regulatory protein α (SIRPα) found on RPMs. Conformational changes to CD47 indicates erythrocyte senescence leading to phagocytosis by fetal RPMs (Murata et al., 2014; Hayes et al., 2020).

Formation of white pulp and the germinal center occurs in the presence of CD209 in humans and SIGN-R1^+^ in murine marginal zone macrophages (MZM) (Steiniger et al., 2007; Endo et al., 2015; Pirgova et al., 2020). After birth, MZM and marginal metallophilic macrophage generation are dependent upon the nuclear liver X receptor (LXR) and function to filter the blood as it is released into the marginal zone (A-Gonzalez et al., 2013). The macrophages act like scavenger cells via scavenger receptors, such as MARCO, which recognize non-opsonised molecules and blood-borne antigens. MARCO also directly binds and mediates the phagocytosis of bacteria such as *Escherichia coli* and *Staphylococcus aureus* and works in conjunction with TLRs to mediate pathogen control (Kellermayer et al., 2014).

Splenic pre-follicular DCs secreting CXCL13 and driving B-cell chemotaxis also contribute to white pulp and marginal zone development after birth (Pirgova et al., 2020). Human fetal spleen cDC1s and cDC2s, observed by 13 PCW, have been observed to induce differentiation of T-regulatory (Treg) cells *in vitro* from adult T-cells. Fetal spleen cDCs also show significantly less pro-inflammatory cytokine production when compared to adult spleen DCs, including increased expression of arginase-2, consistent with the notion of fetal tolerance establishment (McGovern et al., 2017).

### Barrier Organs

#### Lung

Human alveolar macrophages (AMs) express CD64, CD206 and CD163, FABP4, INHBA, SPP1, and MERTK (Mitsi et al., 2018; Morse et al., 2019). AMs reside on the luminal surfaces of the alveoli and are in direct contact with commensal bacteria, inhaled particles, and host-epithelial-derived factors such as surfactants.

In the post-natal steady state, AMs phagocytose excessive surfactant proteins. Mice and humans lacking AMs due to a dysfunction in GM-CSF signaling develop pulmonary alveolar proteinosis as a result of defective surfactant clearance, suggesting a vital function for AMs (Stanley et al., 1994; Robb et al., 1995; Ferretti et al., 2016). Surfactant production in the human fetus has been observed to start between 22 and 24 PCW and needs careful regulation to prevent build up (Hashimoto et al., 2013; Izquierdo et al., 2018).

Interstitial macrophages, present between the airways in the lung tissue interstitium, are involved in tissue remodeling, maintenance, and antigen presentation (van Furth and Cohn, 1968). They interact with DCs to influence airway allergic responses (Bedoret et al., 2009).

#### Gut

The human intestinal tract develops distinct morphological crypt-villus features by 12 PCW (Moxey and Trier, 1978). MPs such as CD103^+^ and CCR7^+^ DCs and macrophages are observed from as early as 14 PCW (Stras et al., 2019). Human intestinal macrophages have been shown to express HLA-DR, CD206, and CD209 (Bujko et al., 2018). Intestinal macrophages assist with epithelial homeostasis during development as they do in post-natal life (D’Angelo et al., 2013; Hoeffel and Ginhoux, 2015). The intestinal epithelium rapidly divides and requires constant and continuous ECM remodeling, which the macrophage provides via Wnt1 signaling and secretion of hepatocyte growth factor (HGF) (D’Angelo et al., 2013; Ortiz-Masia et al., 2014).

Post-natally, intestinal macrophages and DCs can penetrate the epithelium through *trans-*epithelial dendrites (TEDs). TED formation was found to be dependent upon the expression of CX_3_CR1 and the membrane ligand fractalkine (CX_3_CL1). This process allows them to sample and capture luminal bacteria for antigen presentation (Chieppa et al., 2006; Vallon-Eberhard et al., 2006). Intestinal goblet cells assist the transfer of antigens from the intestinal lumen to CD103^+^ DCs (Mazzini et al., 2014; Knoop et al., 2015). These DCs then migrate from the lamina propria (LP) to the mesenteric lymph nodes (MLN) in a CCR7-dependent manner, or within the Peyer’s Patches, into T-cell zones (Jang et al., 2006; Scott et al., 2015).

The human intestinal cDC populations are characterized by their expression of CD103 and SIRPα (Watchmaker et al., 2014). LP DCs are considered tolerogenic and assist with gut homeostasis. CD103^+^ DCs have been observed to metabolize retinoic acid secreted by the liver to induce homing of protective CCR9^+^α_4_^+^β_7_^+^ T and B-cells to the gut (Bakdash et al., 2015; Roe et al., 2017). Other transcription factors, such as transforming growth factor β receptor II (TGFβII) (Ramalingam et al., 2012) and tumor necrosis factor receptor-associated factor 6 (TRAF6) (Han et al., 2013), maintain cDC tolerance of gut microbiota. cDC antigen presentation promotes the generation of forkhead box P3^+^ (FoxP3^+^)-inducible Tregs in the MLN and is key to the development of the symbiotic relationship with microbiota (Esterhazy et al., 2016). Human fetuses start swallowing amniotic fluid from 8 to 12 PCW with data indicating the possible existence of an *in-utero* microbiome in the amniotic fluid and fetal gastrointestinal tract. This evidence thus suggests a potential role for cDC education and tolerance in fetal gut development (Collado et al., 2016; Martinez et al., 2018).

#### Skin

Various populations of MPs reside in the skin, including Langerhans cells (LCs) and dermal DCs. LCs are found in human fetal skin at 4–5 PCW, detected as HLA-DR^+^ and CD1a^+^, harboring a mixed DC/macrophage signature (Foster et al., 1986; Carpentier et al., 2016). LC precursors were observed to acquire CD1c and langerin expression at 9 PCW and grow in number throughout development (Schuster et al., 2009, 2012).

Dermal MPs (macrophages and DCs) are seeded prenatally but are replaced over time by circulating CD14^++^CD16^+^ human monocytes (Chorro et al., 2009; Malissen et al., 2014). CD14^++^CD16^+^ human monocytes can also be recruited to replace LCs when they are unable to self-renew (Chorro et al., 2009; Malissen et al., 2014). In humans, LCs express CD1A, CD11B, CD11C, CD207, and MHC class II. Dermal DCs express lower amounts of CD1A, CD1C, CD11B, CD206, CD209, and MHC class II. Dermal macrophages express CD163 and factor XIIIa (Valladeau et al., 2000; Nestle et al., 2009).

Dermal macrophages also promote the proliferation of fibroblasts in damaged tissue to assist with repair in a TGFα, fibroblast growth factor (FGF), and platelet-derived growth-factor-dependent mechanism (Shook et al., 2018; Etich et al., 2019).

Although human LCs are prenatally derived and share a similar origin with prenatal macrophages, they have additional “DC properties” in their ability to migrate to draining lymph nodes and initiate an immune response (Furio et al., 2010). LCs coordinate a state of immune tolerance in the postnatal skin but can instruct the adaptive immune system when skin integrity is compromised (Seneschal et al., 2012). During the early stages of wound healing, LCs are present as an immune barrier and coordinate with dermal macrophages to promote repair in a fibroblast-dependent manner (Shook et al., 2018; Etich et al., 2019). Little is known about the function of LCs during development, but data suggests they could be involved in ECM remodeling alongside dermal macrophages (Furio et al., 2010). More work is required to fully clarify their role during organogenesis.

Post-natally, dermal DCs function as migratory antigen-presenting cells whilst maintaining tolerance to self-antigens (Haniffa et al., 2012). The equivalent of murine dermal cDC1 in humans is defined as CD141^+^ DCs and co-expresses: XCR1, CADM1, CLEC9A, and TLR3. Skin-draining lymph nodes contain migratory and resident CD141^+^ (Haniffa et al., 2012; Collin and Bigley, 2018).

Developmental macrophage cell programs were recently shown to be co-opted in two common inflammatory skin conditions, psoriasis, and atopic dermatitis (Chorro et al., 2009; Reynolds et al., 2020). These new observations highlight the importance of developmental pathways in inflammatory disease pathogenesis that could be therapeutically targeted.

## Organoid and Organ-On-Chip Platforms to Study Prenatal MPs

Studies on human MPs have focused on *in vitro* culture systems from BM-HSC, peripheral blood monocytes, induced pluripotent stem cells (iPSC) (Caux et al., 1992; Sallusto and Lanzavecchia, 1994; Merad et al., 2002; Poulin et al., 2010; Balan et al., 2018; Kirkling et al., 2018), and *ex vivo* primary MPs isolated from peripheral blood tissues (Haniffa et al., 2013; McGovern et al., 2014; Takata et al., 2017). Such culture systems allow the study of MP interactions with specific cells in the tissues through co-culture. Murine iPSC-derived macrophages (iMacs) can differentiate into microglia in co-culture with iPSC-derived neurons. Murine iMacs can be differentiated into functional TRMs of the lung and brain when transplanted *in vivo* showcasing the remarkable plasticity of MPs (Takata et al., 2017). These studies have provided new insights into the classification and roles of primary MPs but suffer from a failure to allow dissection into how MP ontogeny and functions are shaped by their physiological tissue of residence. Recent developments in human tissue organoid culture systems provide new opportunities to interrogate human MPs. Organoids are 3D culture systems that attempt to model *in vivo* settings by leveraging the intrinsic ability of cells for self-assembly and organization. The most common form of organoid culture, spheroids, organize aggregated cells with or without hydrogen scaffold substrates and aim to replicate three key features of a specific tissue: the spatial distribution of cells, the biochemical environment, and its mechanical environment (Iakobachvili and Peters, 2017; Shanti et al., 2018; Kim et al., 2020). Organoids can facilitate studies on organogenesis, disease pathophysiology, and drug discovery in an *ex vivo* setting (Broutier et al., 2017). A study by Neal et al. (2018) used an air-liquid interface to generate patient-derived tumoral organoids with preserved immune cell types including CD68^+^ CD14^+^ macrophages (Neal et al., 2018). Bourgine et al. (2018) used a perfusion bioreactor system to create a BM organoid with a human osteoblastic environment that supports HSC function (Bourgine et al., 2018).

### Organ-on-Chip Systems

Organoids can replicate organ-level function but may lack key chemical, spatial, or other tissue physico-biomechanical properties distinct from those *in vivo*, e.g., fluid flow, and are labor intensive (Shanti et al., 2018; Jang et al., 2019; Kim et al., 2020). Microfluidic organ-on-chips (OoCs) enable organoids to be cultured in perfused, multiplexed chips that recapitulate tissue-specific mechanical and biochemical parameters at higher throughput (Shanti et al., 2018).

Mononuclear phagocyte migration and its role in development and disease have been studied using OoC models (Biselli et al., 2017; Sasserath et al., 2020). Monocytes and macrophages embedded in OoCs respond to hypoxia and injury-associated signals, such as MCP-1 and IL-6, by migrating down chemokine gradients (Liu et al., 2018; Shanti et al., 2018). These MPs express gene-expression programs similar to those observed to be crucial in development, such as programs involved in angiogenesis (VEGF, COX2, Wnt5a, FGF2), tissue remodeling (MMP9), glucose transport (e.g., solute carrier family 2 member 1), and glycolytic metabolism (enolase 2) (Chou et al., 2018; Shanti et al., 2018; Jang et al., 2019; Sriram et al., 2019). Other OoC models incorporating MPs have been developed for the spleen (Rigat-Brugarolas et al., 2014), skin (Sriram et al., 2019), bone marrow (Chou et al., 2018), liver (Jang et al., 2019), the feto-maternal interface (Richardson et al., 2020), and an inter-connected multi-organ platform (Sasserath et al., 2020).

Sieber et al. (2018) developed a BM-on-a-chip model consisting of two media perfused micro-channels filled with BM progenitor, stromal, and endothelial cells. Cells were embedded in a hydroxyapatite scaffold to mimic the 3D physiology of BM (Sieber et al., 2018). The BM OoC successfully demonstrated key expression programs known to be essential for sustaining the BM HSC niche *in vivo*, with qPCR assays showing upregulation of *nestin, osteopontin, VEGF, angiopoietin 1*, and *fibronectin* expression (Chou et al., 2018; Sieber et al., 2018). CD34^+^ cells isolated from the OoCs could form colony-forming units of erythrocytes, macrophages, granulocyte/macrophage, and granulocyte-erythrocyte-macrophage-megakaryocytes, demonstrating the ability of OoC derived BM HSCs to differentiate into various progenies and maintain functional HSC niches *in vitro* for up to 4 weeks (Sieber et al., 2018). In a separate study by Chou et al. (2018), BM OoCs supported differentiation into myeloid and erythroid-primed lineages, whilst improving maintenance of CD34^+^ progenitors (Chou et al., 2018). Interestingly, post differentiation, myeloid mobilization, and remodeling were also observed across 4 weeks of OoC culture. Selective drug toxicity and recovery from 5-fluoracil and radiation exposures using BM OoCs demonstrated increased biologically mimetic toxicity responses and recoveries compared to their static gel-spheroid counterparts.

Liver OoCs recapitulate the hepatic lobule by patterning hepatocytes and other associated cells via micropillar arrays, which introduce perfusion to maintain functionality over time (Rennert et al., 2015). Liver-on-a-chip platforms have been designed to model and investigate various functions of the liver including metabolism, detoxification, and response to pharmaceutical interventions (Rennert et al., 2015; Groger et al., 2016). Groger et al. (2016) assessed the interaction of circulating monocytes and their ability to trigger tissue repair and the repolarization of Kupffer cells within a polystyrol scaffold embedded in a liver on-a-chip. Inflammation stimulated by TLR1 and 2 agonists and lipopolysaccharide (LPS) in the liver OoC caused the release of pro-inflammatory IL-1β, IL-6, and TNFα within 72 h and anti-inflammatory cytokine IL-10 post 72 h. The OoC exhibited similar responses to livers undergoing sepsis, *in vivo*, which caused hepatocellular dysfunction and cell death. There was also a shift from LPS-induced inflammatory macrophages to regenerative polarization with the introduction of THP-1 monocytes to the system (Groger et al., 2016). These results demonstrate crosstalk of the liver microenvironment and immune system with higher-throughput phenotypic readouts when compared to spheroid cultures (Hotchkiss and Opal, 2010).

A single tissue OoC cannot fully recapitulate physiologically relevant pharmacokinetic properties and toxicity responses across multiple tissues. To achieve crosstalk of organs, long-term multi-OoC cultures have been developed to better mimic these multi-tissue interactions (Zhao et al., 2019). A multi-OoC model comprising cardiomyocytes, skeletal muscle, and the liver was successfully used to study the THP-1 macrophage response to drug and inflammatory stimuli (Sasserath et al., 2020). The multi-OoC model incorporated biological microelectromechanical systems (BioMEMS) to non-invasively measure cardiomyocyte electrical (microelectrode array) and muscle mechanical function (cantilever). Liver function was monitored using biomarker quantification of CYP1A1, 3A4, 2C9, urea, and albumin. The system facilitated a multi-organ response to the drug amiodarone and revealed the selective THP-1 monocyte activation and infiltration in the cardiac OoC due to cytokines released by cardiomyocytes. LPS and IFNγ treatment of the chip system elicited a sepsis-like response characterized by TNFα, IL-6, and CCL5 and decreased cardiac, skeletal muscle, and liver function.

Taken together, these studies demonstrate the effectiveness of OoC systems to study MPs within tissues, as well as their responses to chemical stimuli (Rennert et al., 2015; Groger et al., 2016; Hulsmans et al., 2016; Prabhu and Frangogiannis, 2016; Sieber et al., 2018). However, despite the promising applications of organoids and OoCs, challenges in recapitulating organs to scale with accurate tissue architecture (size, cell number, and distribution) remain (Rennert et al., 2015; Groger et al., 2016).

### The Future of Studying the MP System

To fully establish an atlas of fetal MP populations, studies must go beyond murine models to incorporate biomimetic OoC models with human fetal/embryonic samples (Behjati et al., 2018). Increasingly, international consortia initiatives, such as the Human Development Cell Atlas (HDCA), have leveraged high-throughput, unbiased technologies, such as single-cell RNA sequencing (scRNA-seq), and spatial techniques to create tissue-specific cellular atlases of the developing human (Behjati et al., 2018). In a recent HDCA publication, Popescu et al. (2019) applied scRNA-seq alongside the spatially resolved Hyperion to define the cellular and spatial composition of the human FL and YS, highlighting potential pathways for Kupffer cells to instruct B lineage survival in FL. This data demonstrates the potential for high-throughput omics technologies to inform on the *in vivo* cellular interactions between MPs and their microenvironment.

Organoid culture models have also benefited from the recent surge of accessibility and data generated by omics technologies (Kanton et al., 2019; Lee et al., 2020). Information gleaned from these data repositories can be used to increase the biomimetic capability and complexity of organoid models (Chorro et al., 2009; Haniffa et al., 2012; Popescu et al., 2019). Conversely, the application of high-throughput omics technologies on OoC and organoid models may also inform *in vivo* developmental trajectories and populations (Chorro et al., 2009; Haniffa et al., 2012). In a 2020 study, Lee et al. (2020) developed hair-bearing human skin organoids. These organoids were characterized by scRNA-seq at 1 week and 1-month timepoints and morphologically compared to human 18 PCW fetal skin (Lee et al., 2020). FGF and a BMP-inhibitor were used to induce differentiation of human pluripotent stem cell (hPSC) spheroids into cranial neural crest cell populations, which is key in later assembly of the epidermis. Crucially, differential expression analysis of organoid scRNA-seq data revealed key signaling modulators for self-organization; expression of WNT modulators in the epidermal (*WNT6 and LEF1*) and dermal (*SFRP2*, *TCF4*, *WIF1*, and *APCDD1*) layers may govern the self-assembly and interactions between the respective layers *in vivo*. Furthermore, dermal expression of *FGF7* (Keratinocyte Growth Factor) was also identified as a key driver of epidermal stratification in the organoids (Lee et al., 2020). Similarly, Kanton et al. (2019) recapitulated HDCA scRNA-seq data to reveal the potential and limits of cerebral organoids. Comparison of fetal brain and cerebral organoid scRNA-seq data showed consistent gene-expression patterns of the earliest stage of developmental differentiation trajectories. This could provide an atlas of the budding human brain and offer a baseline for future inclusion of MP cells into cerebral organoid studies (Kanton et al., 2019).

High-throughput scRNA-seq technologies have transformed our understanding of complex cell populations. High-throughput multi-omic approaches can now enable combined scRNAs and cell-surface proteins (CITE-seq) analysis and single nucleus RNA and chromatin accessibility (snATAC-seq) to be performed (Stoeckius et al., 2017). Stephenson et al. (2021) recently used CITE-seq to characterize the cellular immune response to COVID-19 in peripheral blood.

The identification of key modulators driving self-organization and stratification of dermal and epidermal layers provides insight into how factors such as FGF7 and WNT signaling modulators (Lee et al., 2020) may be incorporated into skin OoC models to better regulate organoid cell fates, cell-cell interactions and improve organoid maturation. Data accrued from the HDCA and other international consortia can be leveraged to instruct more complex developmental organoids with the intention of incorporating their associative immune cell and MP populations.

## Conclusion

A thorough understanding of MP contribution to tissue repair and regeneration will have important repercussions for regenerative medicine and therapy. The key roles of MPs in human organogenesis and organ morphogenesis are beginning to be explored and have thus far been reliant on pre-clinical animal models. Several logistical challenges remain to facilitate studies of MPs in human prenatal tissues. Organoids and OoC technologies, in tandem with data generated by emerging omics technologies and international collaborative consortia, provide a new experimental avenue to recapitulate human development and physiology. They have shown early success, but important hurdles remain including successful incorporation of the full complement of immune cells and physiologically relevant vascularization and perfusion of these culture systems.

## Author Contributions

IG and MM contributed equally to literature review, selection, synthesis, and writing. MH contributed to reviewing and editing the manuscript. All authors contributed to the article and approved the submitted version.

## Conflict of Interest

The authors declare that the research was conducted in the absence of any commercial or financial relationships that could be construed as a potential conflict of interest.

## Footnotes

1 https://developmentcellatlas.ncl.ac.uk/MPS_development_review
